# Estimating maximum oxygen uptake of fishes during swimming and following exhaustive chase – different results, biological bases and applications

**DOI:** 10.1242/jeb.246439

**Published:** 2024-05-31

**Authors:** Bernard B. Rees, Jessica E. Reemeyer, Sandra A. Binning, Samantha D. Brieske, Timothy D. Clark, Jeremy De Bonville, Rachel M. Eisenberg, Graham D. Raby, Dominique Roche, Jodie L. Rummer, Yangfan Zhang

**Affiliations:** ^1^ Department of Biological Sciences, University of New Orleans, New Orleans, LA 70148, USA; ^2^ Department of Biology, McGill University, Montreal, QC, Canada H3A 1B1; ^3^ Département de Sciences Biologiques, Université de Montréal, Montréal, QC, Canada H2V 0B3; ^4^ School of Life and Environmental Science, Deakin University, Geelong, Victoria, Australia 3216; ^5^ Department of Zoology, University of British Columbia, Vancouver, BC, Canada V6T 1Z4; ^6^ Department of Biology, Trent University, Peterborough, ON, Canada K9L 0G2; ^7^ Social Sciences and Humanities Research Council of Canada, Ottawa, ON, Canada K1R 0E3; ^8^ College of Science and Engineering, James Cook University, Townsville, QLD 4811, Australia; ^9^ Department of Organismic and Evolutionary Biology, Harvard University, Cambridge, MA 02138, USA

**Keywords:** Respirometry, Aerobic metabolism, Swim tunnel, Exhaustive chase, Individual variation

## Abstract

The maximum rate at which animals take up oxygen from their environment (*Ṁ*_O_2_,max_) is a crucial aspect of their physiology and ecology. In fishes, *Ṁ*_O_2_,max_ is commonly quantified by measuring oxygen uptake either during incremental swimming tests or during recovery from an exhaustive chase. In this Commentary, we compile recent studies that apply both techniques to the same fish and show that the two methods typically yield different mean estimates of *Ṁ*_O_2_,max_ for a group of individuals. Furthermore, within a group of fish, estimates of *Ṁ*_O_2_,max_ determined during swimming are poorly correlated with estimates determined during recovery from chasing (i.e. an individual's *Ṁ*_O_2_,max_ is not repeatable across methods). One explanation for the lack of agreement is that these methods measure different physiological states, each with their own behavioural, anatomical and biochemical determinants. We propose that these methods are not directly interchangeable but, rather, each is suited to address different questions in fish biology. We suggest that researchers select the method that reflects the biological contexts of their study, and we advocate for the use of accurate terminology that acknowledges the technique used to elevate *Ṁ*_O_2__ (e.g. peak *Ṁ*_O_2_,swim_ or peak *Ṁ*_O_2_,recovery_). If the study's objective is to estimate the ‘true’ *Ṁ*_O_2_,max_ of an individual or species, we recommend that pilot studies compare methods, preferably using repeated-measures designs. We hope that these recommendations contribute new insights into the causes and consequences of variation in *Ṁ*_O_2_,max_ within and among fish species.

## Teaser

**Summary:** The two most common methods to estimate the maximum rate of oxygen uptake by fishes give different mean estimates and measures of individual variation. Recognizing that these methods measure different physiological states can enhance their application and interpretation in studies of fish aerobic metabolism.

## Introduction

The maximum rate at which an aquatic animal can take up oxygen from its environment is its *Ṁ*_O_2_,max_ (see Glossary). This rate reflects the upper limit for the extraction and delivery of oxygen to tissues to support aerobic metabolism and it is positively associated with the capacity for sustained aerobic performance (Claireaux et al., 2005; Norin and Clark, 2016). In aquatic ectotherms, especially fishes, *Ṁ*_O_2_,max_ is commonly used as a proxy for maximum metabolic rate (MMR; see Glossary), specifically the aerobic component of MMR (Box 1). In addition, *Ṁ*_O_2_,max_ sets the upper boundary of an animal's scope for metabolic activity (Fry, 1947) or aerobic scope (AS; see Glossary) (Clark et al., 2013). AS reflects the animal's capacity to aerobically fuel activities such as foraging, digestion, predator avoidance, migration, growth and reproduction (Fry, 1947; Priede, 1985; Claireaux and Lefrancois, 2007; Clark et al., 2013). A higher AS, indicating a greater capacity to support these activities, may enhance Darwinian fitness in natural populations (Claireaux and Lefrancois, 2007). Conversely, if AS is reduced by a decrease in *Ṁ*_O_2_,max_ (or an increase in standard metabolic rate, SMR; see Glossary), energetic trade-offs among processes that ‘use’ AS could ensue (Killen et al., 2007; Farrell, 2009; Holt and Jørgensen, 2015; Eliason and Farrell, 2016; Farrell, 2016; Metcalfe et al., 2016). Although the time an animal spends at or near its *Ṁ*_O_2_,max_ is likely to be short and species dependent (Priede, 1977; Farrell, 2016), its activities during this time (e.g. predator avoidance, migration or active foraging) might be disproportionately important to its survival. Hence, a high *Ṁ*_O_2_,max_ and, by extension, an expanded AS are thought to benefit individuals, populations and species.

**Box 1. Is *Ṁ*_O_2_,max_ equal to MMR?**

*Ṁ*_O_2_,max_, the maximum rate at which animals take up oxygen from their environment, is frequently used as proxy of maximum metabolic rate (MMR). Doing so relies upon several seldom-evaluated assumptions (Nelson, 2016; Zhang and Gilbert, 2017), including the following.

(1) Oxygen uptake at the respiratory surface is in steady state with oxygen transport to the tissues and oxygen consumption by the mitochondria. This assumption is probably valid during sustained activity but not necessarily during transitions between activity levels (Farrell and Clutterham, 2003). Hence, measurements of *Ṁ*_O_2__ over brief intervals could fail to reflect tissue oxygen consumption.

(2) Oxidative phosphorylation is the exclusive source of ATP production. At high activity levels, oxygen delivery to skeletal muscle does not match ATP demand, whereupon anaerobic metabolic pathways (e.g. glycolysis) are recruited to supplement oxidative phosphorylation (Burgetz et al., 1998), and *Ṁ*_O_2__ underestimates metabolic rate.

(3) The ratio of ATP production to oxygen consumed (P:O ratio) by mitochondrial oxidative phosphorylation is constant. Because metabolic rate ultimately reflects the rate of ATP turnover, changes in the P:O ratio would alter the relationship between *Ṁ*_O_2__ and metabolic rate. The P:O ratio depends upon the substrate used for mitochondrial metabolism (Hinkle, 2005) and it varies according to physiological state and ecological conditions (Koch et al., 2021; Le Roy et al., 2021; Thoral et al., 2021; Voituron et al., 2022).

In the strictest sense, therefore, metabolic rates (including MMR), are most directly measured as the rate of heat production, i.e. by calorimetry (Regan et al., 2013, 2017). Currently, constraints of calorimeter design generally make this technique impractical for metabolic rate measurements of large, active animals, and it is especially challenging for aquatic animals because of the large heat capacity of water.

Because of these and other caveats, rather than equate *Ṁ*_O_2_,max_ with MMR, it may be more useful (and accurate) to use *Ṁ*_O_2_,max_ for what it is: the maximum rate of oxygen uptake (Nelson, 2016; Zhang and Gilbert, 2017).

**Glossary**

**Aerobic scope (AS)**

A metric that relates *Ṁ*_O_2_,max_ to SMR and is a measure of the capacity for aerobic activities above maintenance levels. Absolute aerobic scope is the difference between these two rates and has the same units as *Ṁ*_O_2__, whereas factorial aerobic scope is the ratio of *Ṁ*_O_2_,max_ to SMR and is unitless.

**Fick equation**

An equation stating that *Ṁ*_O_2__ equals the product of cardiac output and the arteriovenous difference in oxygen content.

**Intermittent-flow respirometry**

A technique for determining *Ṁ*_O_2__ in which the animal chamber alternates between a closed phase, during which the rate of oxygen decline in the chamber is measured, and an open phase, when the chamber is flushed with water to restore oxygen levels and remove metabolic wastes. This technique can be automated and multiplexed, allowing long-term *Ṁ*_O_2__ measurements of several individuals simultaneously (Clark et al., 2013; Svendsen et al., 2016; Killen et al., 2021; Clark, 2022).

***Ṁ*_O_2__**

The rate of oxygen uptake by an animal expressed in units of mass or moles of oxygen per unit time. This term is commonly used for aquatic animals, as opposed to the volume of oxygen taken up (*V̇*_O_2_,max_), which is more common for terrestrial animals.

***Ṁ*_O_2_,max_**

The maximum rate of oxygen uptake, reflecting the maximum oxygen flux across the respiratory surfaces of an animal from its environment.

**Maximum metabolic rate (MMR)**

The theoretical maximum rate of energy expenditure by an animal. In most applications, MMR refers to the maximum aerobic metabolic rate, even though vigorous activity by animals generally relies upon anaerobic processes (e.g. glycolysis) to supplement ATP produced by oxidative phosphorylation (Nelson, 2016).

**Peak *Ṁ*_O_2__**

The highest *Ṁ*_O_2__ determined using a specific protocol. For example, peak *Ṁ*_O_2_,swim_ is the highest *Ṁ*_O_2__ measured during an incremental swim test; whereas, peak *Ṁ*_O_2_,recovery_ is the highest *Ṁ*_O_2__ measured during recovery from exhaustion. This terminology can be extended to other physiological states of elevated *Ṁ*_O_2__ (e.g. digestion; Steell et al., 2019).

**Standard metabolic rate (SMR)**

The minimum rate of energy expenditure by an animal required for maintenance at a given temperature. In fishes, SMR is generally estimated as the lowest stable *Ṁ*_O_2__ of a quiescent, post-absorptive animal when measured over an extended period (18–48 h) and is sometimes referred to as *Ṁ*_O_2_,min_ (Chabot et al., 2016b).

## Methods to determine *Ṁ*_O_2_,max_ in fishes

The Fick equation (see Glossary) states that *Ṁ*_O_2__ (see Glossary) is a function of cardiac output and the difference in oxygen content of arterial and venous blood. Thus, methods to measure *Ṁ*_O_2_,max_ should elicit rates of tissue oxygen consumption that reach the cardiorespiratory limits for oxygen extraction and delivery to the tissues (Jones and Randall, 1978; Rummer and Brauner, 2015; Scott and Dalziel, 2021; Rees et al., 2022). The two most common methods to estimate *Ṁ*_O_2_,max_ in fishes are to use intermittent-flow respirometry (see Glossary) to measure *Ṁ*_O_2__ either (1) as a fish swims at increasing speeds against an imposed current in a swim tunnel or (2) during recovery after an exhaustive chase (Clark et al., 2013; Rummer et al., 2016; Svendsen et al., 2016; Norin and Clark, 2016; Killen et al., 2017). Although we focus on these two methods, higher *Ṁ*_O_2__ values can be attained under other conditions in certain species (see ‘Matching method to biology’, below).

Swim tunnel respirometry measures the *Ṁ*_O_2__ required to support the elevated aerobic metabolism of vigorous, sustained swimming, primarily powered by contraction of red skeletal muscle and largely fuelled by oxidative phosphorylation. The rate of mitochondrial oxygen consumption, and therefore whole-animal *Ṁ*_O_2__ (assuming mitochondrial and organismal oxygen uptake are in a steady state; Box 1), is largely to replenish ATP that is consumed by skeletal muscle cross-bridge cycling and calcium ion regulation (Hoppeler, 2018). In addition, heart rate and ventilation rate are elevated, although ram ventilation reduces the energetic costs of gill ventilation during high-speed swimming in some species (Steffensen, 1985). *Ṁ*_O_2_,max_ is estimated as the highest *Ṁ*_O_2_ _(peak *Ṁ*_O_2__; see Glossary) as the fish swims (i.e. peak *Ṁ*_O_2_,swim_) at increasing water speeds until exhaustion (*U*_crit_ test; Brett, 1964) or during more rapid and dynamic increases in water speed (*U*_max_ test; Clark et al., 2011, Raby et al., 2020). Forced locomotor activity was the first technique used to measure *Ṁ*_O_2_,max_ in fishes (Blazka et al., 1960; Brett, 1964; Steffensen et al., 1984), and it is still considered by many to be the ‘gold standard’ for determining *Ṁ*_O_2_,max_. However, swim tunnel respirometry is time consuming and requires continuous monitoring, which reduces experimental throughput. It is also cumbersome to deploy in remote field conditions, and some fishes either are poor sustained swimmers or cannot be coaxed to perform in a swim tunnel (Clark, 2022).

Because of these challenges, *Ṁ*_O_2_,max_ has also been estimated as the highest *Ṁ*_O_2__ during recovery from an exhaustive chase (hereafter, peak *Ṁ*_O_2_,recovery_) (Soofiani and Priede, 1983; Reidy et al., 1995). Typically, fish are chased by the experimenter in a circular arena and, in some cases, held in air briefly (e.g. 1 min) prior to commencing intermittent-flow respirometry in a chamber with limited volume and minimal water movement (‘static’ chambers). The fish's activity during the chase is supported by both oxidative phosphorylation and anaerobic metabolism (glycolysis and creatine phosphate hydrolysis). During recovery, very little *Ṁ*_O_2__ is used for locomotion, but rather, *Ṁ*_O_2__ remains elevated as a result of reoxygenation of internal oxygen stores (haemoglobin and myoglobin), persistently elevated cardiac activity and ventilation (without the benefit of ram ventilation), re-establishment of pH and ion balance and the clearance of anaerobic end-products (Wood, 1991; Moyes et al., 1992; Scarabello et al., 1992). Although *Ṁ*_O_2__ is expected to decline exponentially after the chase, in some cases peak *Ṁ*_O_2_,recovery_ is not achieved until hours later (Clark et al., 2012; Soofiani and Priede, 1983; Andersson et al., 2020; Brieske et al., 2024). Moreover, the extent to which *Ṁ*_O_2_ _is elevated depends upon how the chase is performed (Reidy et al., 1995; Roche et al., 2013; Zhang et al., 2018), presumably reflecting different degrees of metabolic and cellular disturbance. Nevertheless, the chase method is typically easier and quicker to conduct than swim tunnel respirometry, allowing several individuals to be tested in parallel, thereby increasing experimental throughput (Norin and Malte, 2011; Salin et al., 2016; Reemeyer and Rees, 2020). In addition, the apparatus is more portable, facilitating experiments in remote locations (e.g. Little et al., 2020).

## Rationale and dataset for this Commentary

Despite the differences in the biological processes measured and apparatus used by these two methods, a meta-analysis of data from 121 species of fishes differing in lifestyle (i.e. benthic, benthopelagic, pelagic) did not detect a systematic difference between peak *Ṁ*_O_2_,swim_ and peak *Ṁ*_O_2_,recovery_ (Killen et al., 2017). This conclusion was largely based on values of *Ṁ*_O_2_,swim_ and *Ṁ*_O_2_,recovery_ measured on different individuals and, in some cases, determined in different studies. This conclusion differs from those of a number of studies, many of which used both approaches on the same individuals, showing that peak *Ṁ*_O_2_,swim_ can be substantially higher than peak *Ṁ*_O_2_,recovery_ (Roche et al., 2013; Rummer et al., 2016; Hvas and Oppedal, 2019; Slesinger et al., 2019; Raby et al., 2020; Eisenberg et al., 2024; Brieske et al., 2024). In other species, peak *Ṁ*_O_2_,swim_ is markedly lower than peak *Ṁ*_O_2_,recovery_ (Soofiani and Priede, 1983). Thus, the degree to which these methods yield similar estimates of *Ṁ*_O_2_,max_ remains unclear.

Additionally, several studies have documented that an individual's peak *Ṁ*_O_2__ is repeatable when determined in two or more trials of either swim tunnel respirometry or recovery from an exhaustive chase (Reidy et al., 2000; Marras et al., 2010; Norin and Malte, 2011; Killen et al., 2016; Norin et al., 2016; Reemeyer and Rees, 2020; Brieske et al., 2024). These results are important for two reasons. First, the repeatability of the technique is a measure of its precision. Second, repeatability of peak *Ṁ*_O_2__ over time suggests that it is a stable feature of the individual, and thus potentially subject to evolution by natural selection (see Roche et al., 2016). However, whether an individual's peak *Ṁ*_O_2__ is repeatable when measured by different techniques has received considerably less attention (but see Zhang et al., 2020; Brieske et al., 2024; Eisenberg et al., 2024).

In this Commentary, therefore, we directly compare estimates of peak *Ṁ*_O_2__ when the same individuals were used both in swim tunnel respirometry and during recovery from an exhaustive chase (i.e. using repeated-measures protocols). We asked (1) whether mean estimates of peak *Ṁ*_O_2__ determined by these two approaches were the same, and (2) whether an individual's peak *Ṁ*_O_2__ determined in swim tunnel respirometry was correlated with its peak *Ṁ*_O_2__ after an exhaustive chase (i.e. repeatable across techniques). We extracted data from 10 species representing various fish lineages and habitats, used in 14 experimental trials, conducted over a range of experimental conditions (temperature, salinity and apparatus) (Table S1). All data were published previously (references in Table S1), except for those for pumpkinseed sunfish (*Lepomis gibbosus*). Sunfish were collected, housed and acclimated for 4 weeks to one of three temperatures (20, 25 and 30°C; *n*=12 each) as described in De Bonville et al. (2024). At each temperature, peak *Ṁ*_O_2_,swim_ and peak *Ṁ*_O_2_,recovery_ were determined essentially as described in Binning et al. (2013) and Guitard et al. (2022), respectively. These experiments were approved by Université de Montréal's animal care committee (Comité de déontologie de l'expérimentation sur les animaux; certificate number 22-025).

## Paired comparisons show differences in mean and individual peak *Ṁ*_O_2_ _estimates

**Fig. 1.**
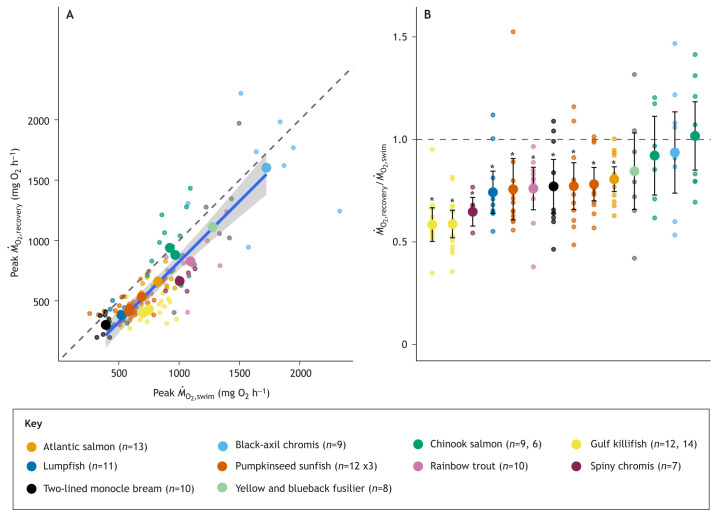
**Agreement between peak rate of oxygen uptake of fishes determined during swim tunnel respirometry (*Ṁ*_O_2_,swim_) and during recovery from an exhaustive chase (*Ṁ*_O_2_,recovery_)**. (A) A scatterplot of peak *Ṁ*_O_2_,recovery_ and *Ṁ*_O_2_,swim_ determined in 14 repeated measures trials with 10 species (the key shows sample sizes per trial; multiple trials with a single species are indicated with corresponding sample sizes). *Ṁ*_O_2__ was normalized to a common temperature (20°C) and body mass (1.0 kg) as in Killen et al. (2017), except that the relationship between *Ṁ*_O_2__, mass and temperature for the current dataset was described by the equation log_10_ *Ṁ*_O_2__=−0.755+1.071×log_10_ body mass+0.0176×temperature. Large coloured circles correspond to the mean for that trial and smaller coloured circles show values for individual fish. The blue line represents the line of best fit (least-squares linear regression) and the grey shaded area indicates the 95% confidence interval (CI). The equation of the line is *Ṁ*_O_2_,recovery_=1.00(*Ṁ*_O_2_,swim_)−172. The 95% CI of the slope is 0.82 to 1.17, and the 95% CI of the intercept is −332 to −11. *r*^2^=0.93. The dashed line shows the line of equality. (B) The ratio of *Ṁ*_O_2_,recovery_ to *Ṁ*_O_2_,swim_. Large coloured circles correspond to the mean for that trial and smaller coloured circles show values for individual fish. The dashed line at 1.0 indicates *Ṁ*_O_2_,recovery_=*Ṁ*_O_2_,swim_. Experimental trials are displayed along the *x*-axis in order of ascending values of this ratio. Error bars show the 95% CI of the mean ratio and asterisks indicate significant differences between *Ṁ*_O_2_,recovery_ and *Ṁ*_O_2_,swim_ (*P*<0.05, paired *t*-tests; see Table S1 for individual *P*-values). See Table S1 for references.

Mean values for peak *Ṁ*_O_2_,recovery_ are plotted against peak *Ṁ*_O_2_,swim_ in Fig. 1A. For this comparison, *Ṁ*_O_2__ was normalized to a common body size (1 kg) and temperature (20°C) (Killen et al., 2017). Although the slope of the relationship was not significantly different from 1.0 (95% confidence intervals, 0.82 to 1.17), the intercept differed from zero (95% confidence intervals, −332 to −11 mg O_2_ h^−1^) and the large majority of individual *Ṁ*_O_2_ _measurements fell below the line of unity, suggesting that peak *Ṁ*_O_2_,recovery_ was less than peak *Ṁ*_O_2_,swim_. This suggestion was reinforced when each individual's peak *Ṁ*_O_2_,recovery_ was expressed as a proportion of its peak *Ṁ*_O_2_,swim_ (Fig. 1B). The mean ratios of peak *Ṁ*_O_2_,recovery_ to peak *Ṁ*_O_2_,swim_ ranged from 0.57 to 1.01 (median 0.765), and for 10 of 14 experimental trials the 95% confidence intervals for this ratio did not overlap one, meaning that peak *Ṁ*_O_2_,recovery_ was significantly less than peak *Ṁ*_O_2_,swim_ (*P*<0.05, paired *t*-tests; see Table S1 for individual *P*-values). Thus, for most species and experimental conditions examined here, peak *Ṁ*_O_2_,recovery_ was substantially lower than peak *Ṁ*_O_2_,swim_ (up to ∼40% lower; median 23.5%). Because these comparisons were paired (i.e. made on the same individuals under defined conditions), this conclusion is not influenced by differences in body size, temperature or other experimental variables.

**Fig. 2.**
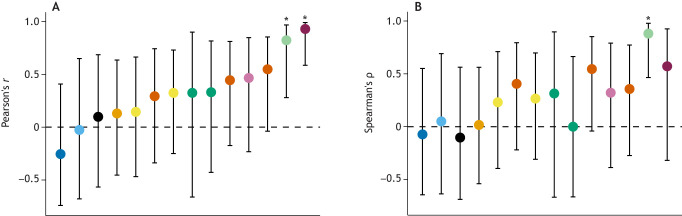
**Repeatability of peak *Ṁ*_O_2__ estimates for individual fish determined during swim tunnel respirometry (*Ṁ*_O_2_,swim_) and during recovery from an exhaustive chase (*Ṁ*_O_2_,recovery_).** (A) Pearson's correlation coefficients (*r*) for the relationship between peak *Ṁ*_O_2_,swim_ and peak *Ṁ*_O_2_,recovery_. Experimental trials are displayed along the *x*-axis in order of ascending *r*. (B) Spearman's rank order correlation coefficients (ρ) in the same species order as in A. Error bars represent the 95% CI of the coefficients and asterisks indicate significant correlations (*P*<0.05; see Table S1 for individual *P*-values). See Fig. 1 for species colour codes and trial sample sizes. See Table S1 for references.

When peak *Ṁ*_O_2_,swim_ and peak *Ṁ*_O_2_,recovery_ were measured in the same individuals, they were generally weakly related or unrelated to one another (Fig. S1). Values for Pearson's correlation coefficients comparing these two metrics within an experiment ranged from −0.25 to 0.93 (median 0.32), and in only two of 14 experimental trials were the correlations between peak *Ṁ*_O_2_,swim_ and peak *Ṁ*_O_2_,recovery_ significant (Fig. 2A; see Table S1 for individual *P*-values). The same pattern arose when each individual's rank within the group was assessed. Spearman's rank order correlation coefficients (ρ) ranged from −0.10 to 0.88 (median 0.29), and in only one experimental trial was the correlation between individual ranks statistically significant (Fig. 2B; see Table S1 for individual *P*-values). Perhaps peak *Ṁ*_O_2__ is simply poorly repeatable for the species included in these analyses. This possibility was directly addressed by Brieske et al. (2024), who found that the repeatability of peak *Ṁ*_O_2_,swim_ of Gulf killifish, *Fundulus grandis*, was high in two trials of swim tunnel respirometry (Pearson's *r*=0.67, *P*<0.05), as was the repeatability of peak *Ṁ*_O_2_,recovery_ in replicate trials of recovery after an exhaustive chase (Pearson's *r*=0.79, *P*<0.01). For the same fish, however, peak *Ṁ*_O_2_,swim_ was unrelated to peak *Ṁ*_O_2_,recovery_ (*r*<0.30, *P*>0.25; Fig. 2A; Table S1, Fig. S1). This study clearly showed that peak *Ṁ*_O_2__ may be consistent when assessed by a given method yet be unrelated across methods. Thus, for the species and contexts studied here, an individual's peak *Ṁ*_O_2_,swim_ has little to no bearing on the same individual's peak *Ṁ*_O_2_,recovery_.

## Experimental design does not explain these differences

Several experimental design considerations affect the accuracy, precision and temporal resolution of *Ṁ*_O_2__ measurements by intermittent-flow respirometry (Clark et al., 2013; Svendsen et al., 2016). Accordingly, we tabulated several features of the experimental designs used in the studies compiled here (Table S1), and asked whether these features were related to either the agreement between mean values or the repeatability of an individual's peak *Ṁ*_O_2__ when assessed by these two approaches (Fig. S2).

Of four attributes related to the species employed in these studies (taxonomic group, habitat, life stage and swimming ability) (Fig. S2A–D), only taxonomic group was related to how well mean peak *Ṁ*_O_2__ estimates agreed between techniques. The four salmonid species, on average, had a higher ratio of peak *Ṁ*_O_2_,recovery_ to peak *Ṁ*_O_2_,swim_ than non-salmonids (*P*=0.047, Mann–Whitney *U*-test; Fig. S2A). This observation is consistent with Little et al. (2020) who found that swim tunnel respirometry and exhaustive chase generally produced similar estimates of peak *Ṁ*_O_2__ in Coho salmon (*Oncorhynchus kisutch*). While the salmonids studied here had somewhat better agreement between peak *Ṁ*_O_2__ determined by these methods than non-salmonids, it is important to note that peak *Ṁ*_O_2_,recovery_ was, nevertheless, statistically lower than peak *Ṁ*_O_2_,swim_ in two of the four trials with salmonids (Fig. 1; Table S1). Also, the repeatability of peak *Ṁ*_O_2__ across these methods was poor for salmonids and non-salmonids alike (Fig. S2A).

Of six attributes related to experimental design (Fig. S2E–J), only the ratio of respirometer chamber volume to fish mass during the chase method was related to the agreement between peak *Ṁ*_O_2__ estimates. When the static chamber used in intermittent-flow respirometry was larger relative to the size of the fish (i.e. chamber volume to fish mass ratio >50), peak *Ṁ*_O_2_,recovery_ and peak *Ṁ*_O_2_,swim_ agreed better than when smaller chambers were used (*P*=0.047, Mann–Whitney *U*-test; Fig. S2I). It could be that larger static chambers allow greater room for fish locomotion, thus elevating peak *Ṁ*_O_2_,recovery_ (Peake and Farrell, 2004). However, this comparison is tempered by three considerations. First, three of four trials with salmonids used larger respirometer chambers, thus confounding chamber volume with taxonomic group (see above). Second, chambers with volume to fish mass ratios <50 are generally recommended for intermittent-flow respirometry because of better temporal resolution and signal to noise ratios (Svendsen et al., 2016). Third, the repeatability of peak *Ṁ*_O_2__ across these methods was equally poor regardless of the chamber volume to fish mass ratio (Fig. S2I).

Other experimental design features reported to influence peak *Ṁ*_O_2_,recovery_ [e.g. time elapsed between the chase and the start of respirometry (Fig. S2G) and whether fish are exposed to air (Fig. S2H)] failed to explain the differences noted in either the ratio of peak *Ṁ*_O_2_,recovery_ to peak *Ṁ*_O_2_,swim_ or the repeatability of an individual's peak *Ṁ*_O_2__. Finally, in nine of 14 experiments, the two methods were applied in a random order (Table S1), suggesting that these results were not biased by factors such as training, fatigue or duration of laboratory maintenance. Therefore, differences in the mean and repeatability of peak *Ṁ*_O_2_ _determined by swim tunnel respirometry compared with exhaustive chase were not obviously related to the species or experimental conditions employed in the studies examined here. Rather, we attribute these differences to the fact that peak *Ṁ*_O_2_,swim_ and peak *Ṁ*_O_2_,recovery_ reflect different physiological states, each with their own underlying determinants and ranges of variation among individuals.

## Improving methods to measure peak *Ṁ*_O_2__

Because peak *Ṁ*_O_2__ is dynamic and context dependent, devices and analytical techniques must be able to capture transiently elevated rates. Given the high sampling frequency of oxygen sensors and the development of ‘rolling’ or ‘sliding-window’ regressions in analytical software, it is now possible to evaluate multiple measurement intervals and select the shortest interval that captures the highest *Ṁ*_O_2__ without sacrificing precision (Box 2; Zhang et al., 2019, 2020; Little et al., 2020; Prinzing et al., 2021). Thus, ‘rolling’ regression is more likely to capture transiently elevated *Ṁ*_O_2__ than determinations made over longer measurement intervals, and it should be incorporated into analyses whose goal is to estimate peak *Ṁ*_O_2__.

**Box 2. Increasing the temporal resolution of *Ṁ*_O_2__ determination**

The time required to accurately determine *Ṁ*_O_2__ during intermittent-flow respirometry depends upon the rate of oxygen uptake, the sensitivity of the measurement device and respirometer design (Svendsen et al., 2016). Commonly, *Ṁ*_O_2__ is measured over intervals of 3–20 min, which results in an average rate over the entire interval. However, *Ṁ*_O_2__ is dynamic during swimming or recovery from an exhaustive chase, and accurate estimates of peak *Ṁ*_O_2__ require a technique that confidently captures the highest rate. In ‘sliding window’ or ‘rolling’ regression, the decline in oxygen is determined over the shortest sampling window that achieves an adequate level of precision (Zhang et al., 2019; 2020; Little et al., 2020; Prinzing et al., 2021). This is done by fitting linear regressions to intervals of increasing duration, and for each duration, the slopes over all possible intervals are determined (i.e. each advancing by the sampling frequency of the sensor) (https://github.com/boennecd/rollRegres).

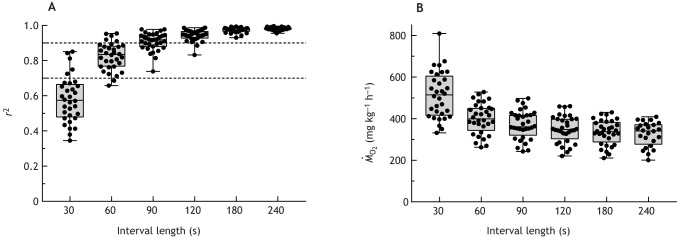

In the example shown above, dissolved oxygen concentrations were recorded during recovery from an exhaustive chase of the Gulf killifish, *Fundulus grandis* (two trials, *n*=16 each) (Brieske et al., 2024). The rate of oxygen decline was determined over all possible intervals ranging from 30 to 240 s, each advancing by 1 s (e.g. 211×30 s intervals; 1–30, 2–31…211–240). For each interval length, the single highest slope was used to determine *Ṁ*_O_2__. A 90 s sampling window had a median *r*^2^>0.90 (see figure, panel A, upper dotted line) and all trials had *r*^2^>0.70 (panel A, lower dotted line). The mean *Ṁ*_O_2__ measured over 90 s was 14% higher than that determined over the entire 4 min period (panel B). Shorter intervals yielded higher estimates of *Ṁ*_O_2__, but they were less precise. At very short intervals (≤30 s), the range of *Ṁ*_O_2_ _estimates may include negative values (Zhang et al., 2020), clearly indicating that shorter measurement intervals can yield spurious estimates. Ultimately, the choice of the appropriate sampling interval strikes a balance between precision and the ability to capture transiently elevated *Ṁ*_O_2__.

Another innovation is to modify a static respirometer chamber by introducing a chasing device, allowing the fish to be motivated to swim vigorously while *Ṁ*_O_2__ is simultaneously recorded (Norin and Clark, 2016; Zhang et al., 2019, 2020). Such modification allows determination of peak *Ṁ*_O_2__ during the chase itself (*Ṁ*_O_2_,chase_). When this modification was combined with ‘rolling’ regression, peak *Ṁ*_O_2_,chase_ of juvenile rainbow trout (*Oncorhynchus mykiss*) was higher than *Ṁ*_O_2_,recovery_ but not different from peak *Ṁ*_O_2_,swim_ of the same individuals (Zhang et al., 2020). Moreover, peak *Ṁ*_O_2_,chase_ and peak *Ṁ*_O_2_,swim_ of individual fish were correlated (i.e. repeatable; Pearson's *r* and Spearman's ρ ≥0.77; *P*<0.05; Zhang et al., 2020), whereas neither was significantly correlated with *Ṁ*_O_2_,recovery_. In contrast, a study on juvenile barramundi (*Lates calcarifer*) showed that *Ṁ*_O_2_,chase_ was not as high as *Ṁ*_O_2_,recovery_ measured immediately post-chase (Norin and Clark, 2016, 2017). Clearly, more validation is required, but using a modified ‘chase respirometer’ might offer researchers a tool that is higher throughput, lower cost and more portable than traditional swim tunnel respirometry, while also providing estimates of peak *Ṁ*_O_2__ that may be similar to *Ṁ*_O_2_,swim_ in mean and repeatability.

## Matching method to biology

Because swim tunnel respirometry and exhaustive chase protocols measure different physiological states, it follows that they are not equivalent methods to estimate *Ṁ*_O_2_,max_ in fishes. Rather, each method is useful to explore a different set of biological questions and, potentially, better suited for a given species.

For questions related to the aerobic costs of locomotion, swim tunnel respirometry is the obvious choice. Swim tunnels and swimming protocols can be modified to accommodate fishes with different swimming styles and abilities (Priede and Holliday, 1980; Van den Thillart et al., 2004; Clark et al., 2011; Rummer et al., 2016). However, some species are not strong, sustained swimmers, and using swim tunnel respirometry to determine the *Ṁ*_O_2_,max_ of such species might not be appropriate. We also acknowledge that even for strong, sustained swimmers, the dimensions of swim tunnels might constrain activity and underestimate a fish's swimming capacity in more naturalistic settings (Peake and Farrell, 2006; Kern et al., 2017).

An exhaustive chase protocol could help define the metabolic costs of recovering from vigorous activity such as predator avoidance or capture. This could be more ecologically relevant than the cost of sustained swimming in certain conservation or fisheries studies. For example, following the chase with a period of air exposure was originally developed as a way of mimicking the stress and handling of catch-and-release sport fishing (Donaldson et al., 2010; Clark et al., 2012).

It is also possible that *Ṁ*_O_2_,max_ is reached during states other than sustained swimming or recovery from an exhaustive chase. Ingestion of a meal brings about an increase in *Ṁ*_O_2__, which is thought to reflect the cost of food handling, breakdown, assimilation and somatic growth (Brett and Groves, 1979; Goodrich et al., 2024). The magnitude of the post-prandial *Ṁ*_O_2__ depends upon the species of fish, the quantity and quality of food consumed, and other factors (e.g. temperature; Chabot et al., 2016a). In some circumstances, the post-prandial *Ṁ*_O_2__ may approach, or even exceed, the *Ṁ*_O_2__ measured during swimming or after an exhaustive chase, especially for poor-swimming predatory fishes that consume large meals (i.e. ambush predators; Soofiani and Hawkins, 1982; Fu et al., 2009; Steell et al., 2019). Yet other species might achieve their highest *Ṁ*_O_2_ _during spontaneous activity as a result of light changes associated with photoperiod (Andersson et al., 2020).

## Conclusions and recommendations for future studies

Using paired comparisons of *Ṁ*_O_2__ determined for a diverse group of fishes measured under an array of experimental conditions, we show that peak *Ṁ*_O_2__ depends upon the method used. This is true for mean values of peak *Ṁ*_O_2_ _determined for a group of individuals, as well as for the repeatability of an individual's peak *Ṁ*_O_2__. These results reinforce the need to carefully consider the biological context of experiments that measure peak *Ṁ*_O_2__ (Roche et al., 2013; Clark et al., 2013; Norin and Clark, 2016; Farrell, 2016; Rummer et al., 2016; Raby et al., 2020) and lead to several recommendations for studies of elevated aerobic metabolism in fishes (Box 3).

**Box 3. Recommendations for studying peak *Ṁ*_O_2__ in fishes**

We offer the following recommendations to consider when designing or interpreting studies of elevated aerobic metabolism in fishes.

(1) Different methods of elevating a fish's *Ṁ*_O_2__ will likely yield different estimates of peak *Ṁ*_O_2__ and a different order of individual *Ṁ*_O_2__ values within a group. Thus, one should use the method for determining peak *Ṁ*_O_2_ _that best suits the biology of the organism and the question of interest.

(2) If the goal of a study is to estimate the ‘true’ *Ṁ*_O_2_,max_ for an individual or a species, we recommend comparing peak *Ṁ*_O_2__ determined by different methods. Ideally, such comparisons would employ repeated measurements on the same individuals, randomized trial order and adequate sample sizes (ideally *n*≥20) to robustly discriminate among methods.

(3) When relating the peak *Ṁ*_O_2__ to other traits measured on the same individuals or species, these relationships may depend upon the method used to determine peak *Ṁ*_O_2_ _(Lawrence et al., 2023_;_ Brieske et al., 2024). If peak *Ṁ*_O_2_,swim_ and *Ṁ*_O_2_,recovery_ reflect different physiological states, then the strength of their correlation to other behavioural, anatomical, biochemical and genetic traits will almost certainly differ.

(4) Follow current recommendations for designing and conducting respirometry experiments (e.g. Clark et al., 2013; Svendsen et al., 2016; Killen et al., 2021; Clark, 2022), and include ‘rolling’ regression with a conservative minimum sampling window to estimate dynamic changes in peak *Ṁ*_O_2__ (Box 2; Zhang et al., 2020).

(5) Finally, employ consistent terminology that accurately reflects the method used to elevate *Ṁ*_O_2__ (e.g. peak *Ṁ*_O_2_,swim_, peak *Ṁ*_O_2_,recovery_).

We hope that these recommendations will be integrated into the determination of peak *Ṁ*_O_2__ in future studies of fish physiology, behavioural ecology, conservation and management. Such studies might explore how certain biotic or abiotic variables differentially affect peak *Ṁ*_O_2__ measured by diverse methods. For example, exposure to elevated temperature might have different effects on peak *Ṁ*_O_2_,swim_ and peak *Ṁ*_O_2_,recovery_ or the ranking of individual *Ṁ*_O_2__ determined by these two methods. Such outcomes would suggest that the underlying physiological processes differ in their thermal sensitivities, which could have implications for a fish's capacity for sustained swimming versus recovery from burst swimming in the context of climate warming (Clark et al., 2017; Johansen et al., 2021). Furthermore, parasites that impair sustained swimming (e.g. Palstra et al., 2007) might alter the group mean and individual variation of peak *Ṁ*_O_2_,swim_ but not peak *Ṁ*_O_2_,recovery_. It would also be valuable to compare the repeatability of peak *Ṁ*_O_2_,swim_ and peak *Ṁ*_O_2_,recovery_ over the lifespan of individuals to assess the influences of ontogeny (e.g. Downie et al., 2023) and acclimation to changing environments (e.g. Auer et al., 2018; Reemeyer and Rees, 2020).

By appreciating that these methods measure different biological processes and address different biological questions, we hope to enhance our understanding of both the biology of fishes and the impacts of human-induced changes to aquatic habitats.

## Supplementary Material

10.1242/jexbio.246439_sup1 Supplementary information

Table S1.
