# Supplementary material for: Estimating maximum oxygen uptake of fishes during swimming and following exhaustive chase – different results, biological bases and applications
Source: J Exp Biol. 2024 May 31;227(11):jeb246439. doi: 10.1242/jeb.246439 (PMC11152165; doi:10.1242/jeb.246439)
Supplement: Supplementary information [file jexbio-227-246439-s1.pdf]

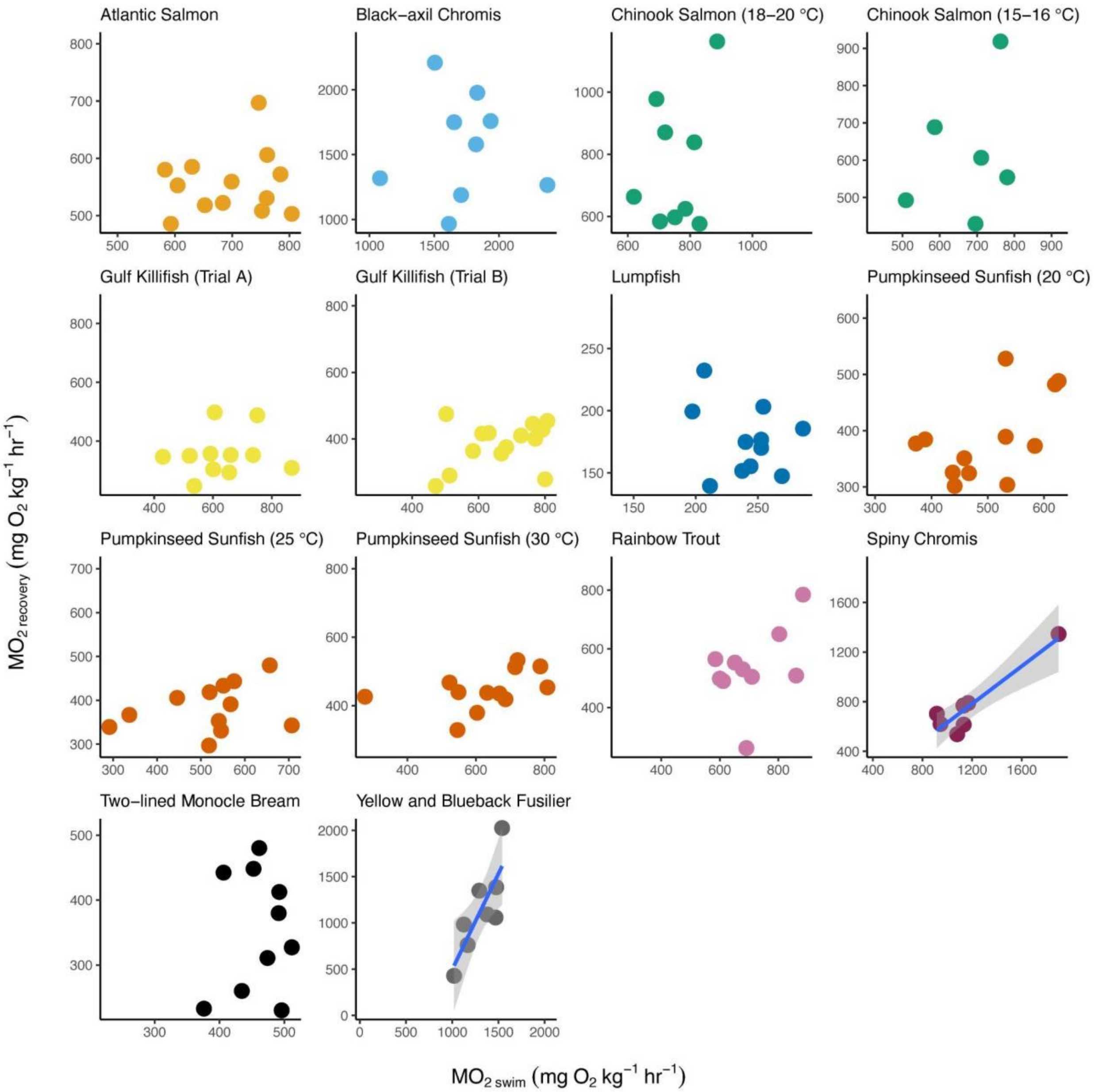


**Fig. S1. Scatterplots of peak $\dot{M}O_2$ determined during swim tunnel respirometry ($\dot{M}O_{2swim}$) versus peak $\dot{M}O_2$ during recovery from an exhaustive chase ($\dot{M}O_{2recovery}$) for each dataset used in this study.** Symbols are colored according to species (indicated in the panel title) consistent with Figs. 1 and 2. Panel titles also indicate if a given species was used in multiple repeated-measures trials. In cases where the Pearson's correlation coefficient between $\dot{M}O_{2swim}$ and $\dot{M}O_{2recovery}$ was significant ($P < 0.05$), blue lines represent lines of best fit (least-squares linear regression) 95% CI are shown.

Journal of Experimental Biology • Supplementary information

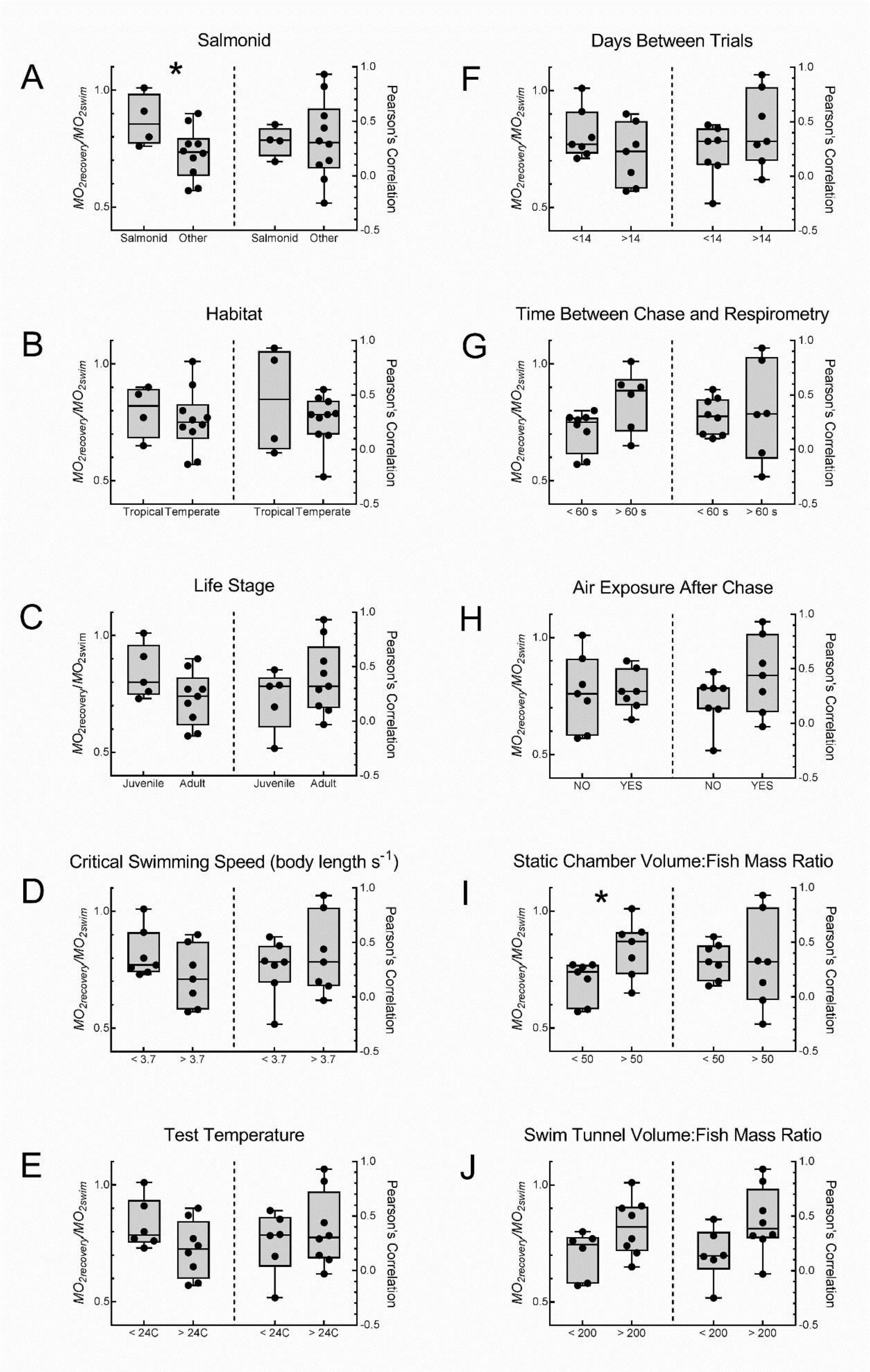


Journal of Experimental Biology • Supplementary information

**Fig. S2. The influence of biological (A-D) and experimental factors (E-J) on the agreement between mean peak $\dot{M}O_2$ and the repeatability of individual $\dot{M}O_2$ determined during swim tunnel respirometry ($\dot{M}O_{2swim}$) and during recovery after an exhaustive chase ($\dot{M}O_{2recovery}$).** Data from Table S1 were divided into groups based upon the following criteria: (A) Fish taxonomic group (salmonids or non-salmonids); (B) Habitat (tropical or temperate); (C) Life stage (juvenile or adult); (D) Swimming ability, as judged by critical swimming speed (< 3.7 body lengths $s^{-1}$ or > 3.7 body lengths $s^{-1}$); (E) Test temperature (< 24°C or > 24°C); (F) Days between trials (< 14 days or > 14 days); (G) Time between the end of chasing and the start of respirometry (< 60 s or > 60 s); (H) Whether fish were exposed to air after the chase and prior to respirometry (no or yes); (I) The ratio of the static respirometer chamber volume in ml to fish body mass in g (< 50 or > 50); (J) The ratio of the swim tunnel volume in ml to fish body mass in g (< 200 or > 200). Some groups were determined by the species used (e.g., taxonomic group, habitat) and some groups were formed to achieve approximately equal numbers in two groups (e.g., critical swimming speed). For each panel, the left y-axis shows the ratio of $\dot{M}O_{2recovery}$ to $\dot{M}O_{2swim}$ and the right y-axis shows the value of Pearson's correlation coefficient (note the different scales). Mann-Whitney tests were performed to assess whether the ratio of $\dot{M}O_{2recovery}$ to $\dot{M}O_{2swim}$ or Pearson's correlation coefficients differed between groups. Asterisks show significant differences between groups (both $P$ = 0.047).

**Table S1.**

Available for download at
https://journals.biologists.com/jeb/article-lookup/doi/10.1242/jeb.246439#supplementary-data

Journal of Experimental Biology • Supplementary information
